# Chronic dizziness in older adults: Disrupted sensorimotor EEG beta oscillations during postural instability

**DOI:** 10.1016/j.clinph.2025.03.032

**Published:** 2025-03-27

**Authors:** Toby J. Ellmers, Richard Ibitoye, Patricia Castro, Elmar C. Kal, Diego Kaski, Adolfo M. Bronstein

**Affiliations:** a Department of Brain Sciences, Imperial College London, Charing Cross Hospital, London, UK; b Department of Clinical and Movement Neurosciences, University College London, London, UK; c Universidad del Desarrollo, Escuela de Fonoaudiología, Facultad de Medicina Clínica Alemana, Santiago, Chile; d Centre for Cognitive and Clinical Neuroscience, Department of Health Sciences, College of Health, Medicine, and Life Sciences, Brunel University London, Uxbridge, UK

**Keywords:** Aging, Small vessel disease, Vestibular, Balance, EEG

## Abstract

**Objective:**

Chronic dizziness is common in older adults, yet frequently occurs without a clear cause (‘idiopathic dizziness’). Patients experience subjective unsteadiness with minimal objective imbalance, potentially related to small vessel disease. Here we examine the hypothesis that this syndrome is associated with disrupted cortical processing of postural instability.

**Methods:**

EEG and postural sway were recorded in 33 older adults with chronic, idiopathic dizziness (Age, Mean = 77.3 years, SD = 6.4, 61 % female) and 25 matched controls (Age, Mean = 76.9 years, SD = 6.0, 56 % female). EEG was time-locked to spontaneous instances of postural instability and analysed via time–frequency decomposition.

**Results:**

Significant between-group differences in EEG were observed during the early phase of postural instability (*p* < 0.05, cluster-corrected). Whilst controls exhibited broadband increase in EEG power across sensorimotor areas, dizzy patients displayed suppressed beta activity (19–24 Hz). Contrary to predictions, these differences did not relate to small vessel disease markers (*r*s < 0.05, *p*s > 0.720) but to fear of falling (*r* = -0.44, *p* = 0.001).

**Conclusions:**

Previous work implies that suppressing cortical beta enhances the relay of sensory information. We therefore propose that the modulation in beta EEG observed in patients reflects an anxious, top-down strategy to increase sensitivity to instability, which paradoxically causes persistent feelings of subjective imbalance.

**Significance:**

These results identify associations between idiopathic dizziness and disrupted sensorimotor beta activation during postural instability. Cortical beta during imbalance may be a possible biomarker of chronic, idiopathic dizziness in older adults and/or fear of falling.

## 1 Introduction

Dizziness is common in older adults (Colledge et al., 1994). For up to 50 % of patients, the cause of dizziness cannot be identified through detailed clinical neuro-otological testing (Ahmad et al., 2015; Castro et al., 2024; Ibitoye et al., 2021). Recent work suggests that these symptoms may instead be a consequence of altered balance perception resulting from subtle disturbances to postural brain networks (likely due to small vessel disease) and associated changes in top-down balance control (Castro et al., 2024; Ibitoye et al., 2021). As a result, these individuals experience vague feelings of subjective unsteadiness in the absence of any major disruption to balance (Castro et al., 2024; Ibitoye et al., 2021). The cortical origin of this symptom is reflected in the significant overlap between this syndrome (Bronstein et al., 2024) and the presence of cognitive impairment in dizzy older adults (Felfela et al., 2024).

As the bodily centre-of-mass is positioned slightly in-front of the ankle joint, quiet stance is characterised by frequently arrested small fall-like events. Regulating these “micro-falls” (and thus preventing a true loss of balance) has been shown to rely on cortical input. Specifically, Nakamura et al. (2023) reported an initial burst of EEG beta frequency band activity in young adults that was time-locked to the beginning of the micro-fall. This was then followed by a marked suppression in beta activity once the micro-fall had been arrested. Cortical beta (~13–30 Hz) is believed to play a top-down inhibitory role across sensorimotor function (Barone and Rossiter, 2021; Engel and Fries, 2010). Indeed, increased beta activity is associated with both motor (Jana et al., 2020; Swann et al., 2009, 2012; Wagner et al., 2018) and perceptual inhibition (Mirdamadi et al., 2024; Shin et al., 2017). Nakamura et al. (2023) therefore interpreted their observed time-locked patterns of beta activation during instability as reflecting a “stop command” to inhibit the micro-fall and/or the active monitoring of re-afferent sensory feedback.

Yet, although frequently occuring, these micro-falls typically occur outside of conscious awareness (Fitzpatrick et al., 1992). Here, we test the hypothesis that chronic dizziness in older adults may be associated with cortical over-responsiveness to micro-fall events; resulting in these individuals becoming consciously aware of small, regularly occurring postural instability (Ellmers and Kal, 2024).

## 2 Methods

### 2.1 Participants

We describe a subset of participants reported previously in this journal (Ibitoye et al., 2021). All participants with EEG and balance data from this previous work were analysed, leaving 33 older adults with idiopathic dizziness (‘Patients’; Age, Mean = 77.3 years, SD = 6.4, 60.6 % female) and 25 non-dizzy individuals matched closely on age and gender (‘Controls’; Age, Mean = 76.9 years, SD = 6.0, 56.0 % female; see Table 1). Patients had ‘dizziness’ as their primary reason for consultation. We found no neurological (including brain MRI), cardio-vascular or vestibular function abnormalities (including video head impulse test and/or caloric testing, thus ruling out presbyvestibulopathy (Agrawal et al., 2019)). Similarly, no patient met the Barany Society diagnostic criteria for Persistent Postural-Perceptual Dizziness (Staab et al., 2017). Symptoms were long-standing (*Mean* = 6 ± 5 [*SD*] years), not precipitated by a pre-existing vestibular disorder, and no alternative diagnoses emerged after at least 6 months follow-up. Healthy controls without a history of dizziness (or neuro-otological dysfunction) were recruited from local community networks. Written informed consent was obtained and the study was approved by the North-East-York Research Ethics Committee.

Participants completed questionnaires to assess their concerns (i.e. fear) about falling (Short Falls Efficacy Scale—International (FES-I) (Kempen et al., 2008)), anxiety and depression (Hospital Anxiety and Depression Scale (HADS) (Zigmond and Snaith, 1983)) and dizziness handicap (Dizziness Handicap Inventory (DHI) (Jacobson and Newman, 1990)). The Short Physical Performance Battery was used to assess functional balance, lower extremity strength and physical function (Guralnik et al., 1994), whilst ankle sensory thresholds to vibration (64 Hz) were measured using a quantitative tuning fork (0–8, lower is worse (Bergin et al., 1995)). Finally, participants underwent a research MRI scan to determine white matter hyperintensity (WMH) load, number of lesions, and mean fractional anisotropy (FA) in white matter skeleton (see (Ibitoye et al., 2022) for further details).

### 2.2 Protocol

Participants wore a 32-channel EEG cap (Waveguard™ cap, ANT Neuro, The Netherlands; sampling at 1250 Hz) according to the 10–20 system, with a 33rd reference electrode located 10 % anterior to Fz. Participants performed four 120 s trials of quiet standing on a solid surface with their eyes closed, barefoot, and with feet shoulder-width apart. After each trial, participants rated their perceived (i.e., subjective) postural instability on a visual analogue scale (0 = ‘completely stable’, 10 = ‘so unstable I would fall’ (Castro et al., 2019)).

### 2.3 Identifying micro-fall events

Postural displacement during each trial was recorded via a Fastrak electromagnetic linear motion tracking device (Polhemus, VT) attached firmly to the occiput sampling at 1250 Hz. Fastrak data were sampled simultaneously alongside EEG signals via an auxiliary channel into the EEG amplifier, ensuring accurate synchronisation between signals. Postural displacement outcomes recorded from the head are highly correlated with those recorded via centre of pressure during quiet stance, with R2 values of >0.90 (Sylcott et al., 2021). Displacement data were low-pass filtered offline at 5 Hz and the mean position was then subtracted from the signal. This data was used to estimate sway displacement and sway velocity within the anterior-posterior direction. In line with previous work (Nakamura et al., 2023), micro-fall events were identified by large peaks in forward sway velocity. We estimated 3 SDs of the sway velocity data in each of the four trials and then averaged this to identify a subject-specific ‘instability threshold’. Velocity peaks that exceeded this threshold were identified using a custom MATLAB script. Only peaks that did not occur within two seconds of a larger peak were identified which ensured that our analysis was restricted to the most prominent micro-fall events. The first 10 s of each trial was discarded to avoid transient effects. The instability thresholds used to classify a micro-fall was similar in both Controls (*Mean* = 32.15 mm/s, *SD* = 9.06 mm/s) and Patients (*Mean* = 38.04 mm/s, *SD* = 15.88 mm/s; *Z* = -0.98, *p* = 0.326). The number of analysed micro-fall events (and thus EEG epochs; see below) were comparable between Controls (*Mean* = 70.7, *SD* = 11.8) and Patients (*Mean* = 71.5, *SD* = 15.1; *t* = -0.22, *p* = 0.829).

### 2.4 EEG pre-processing and analysis

Participant EEG data was first concatenated across individual trials. It was then resampled to 500 Hz and pre-processed in MATLAB using the Harvard Automated Processing Pipeline for Electroencephalography (HAPPE) pipeline (Gabard-Durnam et al., 2018). This consisted first of 50 Hz line-noise removal using CleanLine, followed by a 1–100 Hz bandpass filter using the EEGLab (Delorme and Makeig, 2004) ‘pop_eegfiltnew’ function (a zero-phase Hamming-windowed-sinc finite impulse response filter). Next, bad channels were identified using EEGLab’s Clean Rawdata algorithm, followed by an additional power spectrum-based bad channel detection step. HAPPEE utilises a two-step approach for addressing artifacts: wavelet-thresholding, followed by traditional independent component analysis (ICA). Briefly, wavelet-thresholding identifies time- and frequency-localised artifacts. This allows for the subsequent removal of specific contaminated frequencies from the pre-thresholded timeseries in which they occur. ICA was then applied using the Runica EEGLab plugin and automated component rejection was implemented using the Multiple Artifact Rejection Algorithm (Winkler et al., 2014). A similar number of components were removed in Controls (*Mean* = 4.6, *SD* = 4.5) and Patients (*Mean* = 4.5, *SD* = 3.4; *Z* = -0.39, *p* = 0.698). Finally, bad channels were interpolated, and then data were re-referenced to the average and epoched to ±1.5 s around micro-fall peaks.

### 2.5 EEG time-frequency analysis

We focused our analyses on electrode Cz as previous research has shown the cortical response to micro-falls to be largest over this electrode (Nakamura et al., 2023; Zaback et al., 2023). Event-related spectral perturbation (ERSP) was calculated via trial-by-trial convolution with complex Morlet wavelets. We used 33 frequencies linearly spaced between 3–45 Hz, with wavelets logarithmically spaced from 4—12 cycles. Unlike traditional ERSP analyses (whereby a stimulus is presented following a period of baseline activity), analysing discrete micro-falls during quiet stance renders the identification of a ‘true’ baseline period difficult. We therefore used the entire 3-second epoch (excluding the first and last 150 ms to avoid edge artefacts) as the ‘baseline’ in which to normalise ERSP data to (as per (Nakamura et al., 2023)), with ERSP data presented in decibels (dB). As mean micro-fall events for both groups consisted of a 400 ms initial fall followed by a 400 ms recovery after which velocity returned to a zero-point (see sway velocity grand averages; Fig. 2), ERSP analyses are presented within this time-window. All analysis steps were performed using custom-written scripts for MATLAB (adapted from (Cohen, 2014)). All analyses were performed blinded to participant grouping.

### 2.6 Statistical analysis

Objective and subjective (perceived instability) balance outcomes were compared between groups using Mann-Whitney U tests. We first calculated within-group changes in ERSP data (compared to baseline) using non-parametric permutation testing (1000 iterations). Only statistically significant differences in ERSP for each group are presented in Fig. 2 (*p* < 0.05). We next calculated between-group differences in ERSP data using non-parametric permutation testing (1000 iterations), followed by cluster-based correction to address the issue of multiple comparisons. We adopted the data-driven thresholding approach (as described in (Cohen, 2014)). Briefly, this involved identifying the largest statistically significant cluster (based on supra-thresholded pixels when set to *p* < 0.05) from each initial non-parametric permutation test. A distribution of the largest supra-threshold clusters that can be expected under the null hypothesis was then generated and only clusters that are larger than the 95th percentile of this distribution were retained (i.e., corrected significance level set to *p* < 0.05). Any areas of between-group significance were then averaged across the relevant time–frequency domains and correlated (across the whole sample) with concerns about falling (FES-I), anxiety (HADS-A), white matter MRI outcomes and ankle vibration perceptual thresholds, to better understand the mechanisms contributing to any between-group EEG differences.

## 3 Results

### 3.1 Objective and subjective balance

**Fig. 1:**
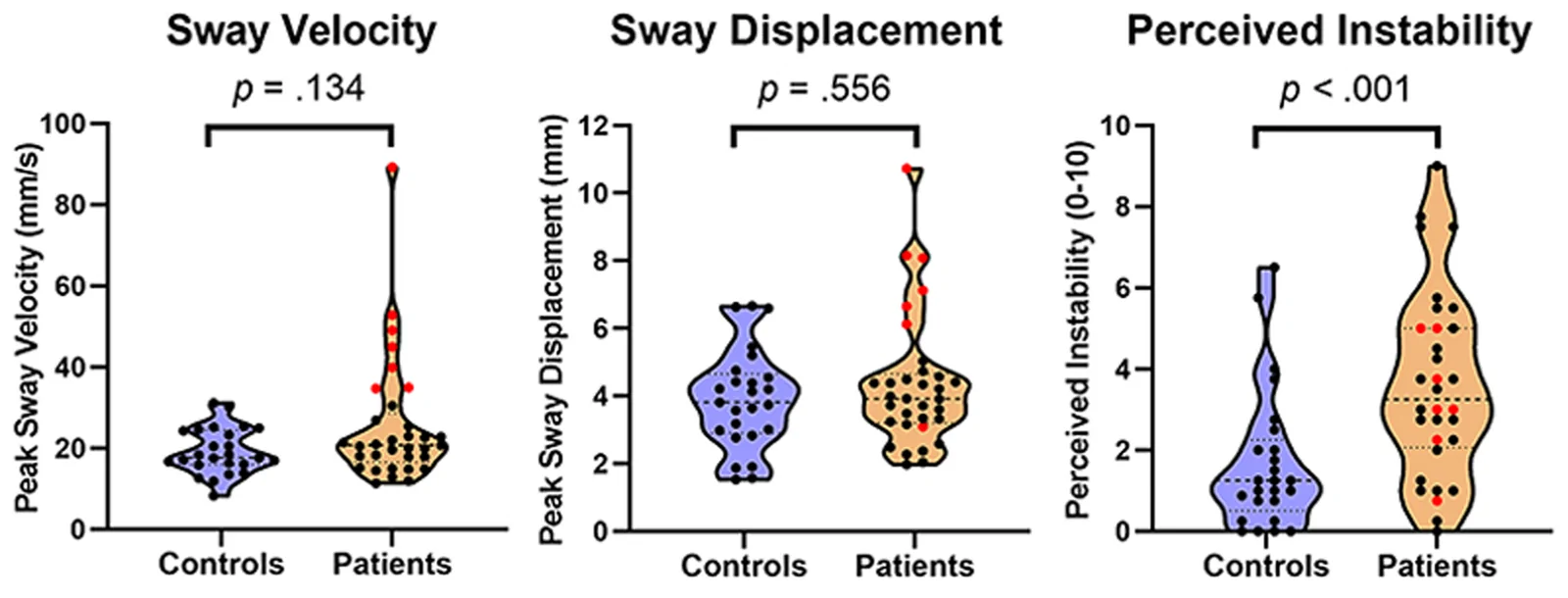
Violin plots (dashed line reflects the median and dotted lines reflect the quartiles) and individual datapoints for peak sway velocity (left panel) and peak sway displacement during micro-fall events (middle panel), and self-reported perceived instability averaged across the task (right panel). Note, the seven ‘unstable’ participants (whose peak sway velocity fell outside that of the Control participants) are highlighted with red individual datapoints in each figure.

As illustrated in Fig. 1, neither peak sway velocity (*Z* = -1.50, *p* = 0.134) nor displacement (*Z* = -0.59, *p* = 0.556) during micro-fall events significantly differed between groups. However, seven patients did appear somewhat more unstable (see red individual datapoints in Fig. 1). Similarly, there was a lack of between-group difference for overall sway velocity (Controls: *Mean* = 8.32 mm/s, *SD* = 2.33; Patients: *Mean* = 9.83 mm/s, *SD* = 4.11; *Z* = -0.90, *p* = 0.367) or displacement (RMS; Controls: *Mean* = 9.67 mm, *SD* = 2.85; Patients: *Mean* = 11.44 mm, *SD* = 4.44; *Z* = −1.52, *p* = 0.130) averaged across the entire 120-second trials. Despite this lack of overall objective difference in balance, Patients reported feeling significantly more unstable (*Z* = −3.38, *p* < 0.001; Fig. 1). Importantly, this significant difference remained when removing the seven ‘unstable’ Patients (red datapoints in Fig. 1) from this analysis (*Z* = −2.98, *p* = 0.003).

### 3.2 EEG Time-Frequency analysis

**Fig. 2:**
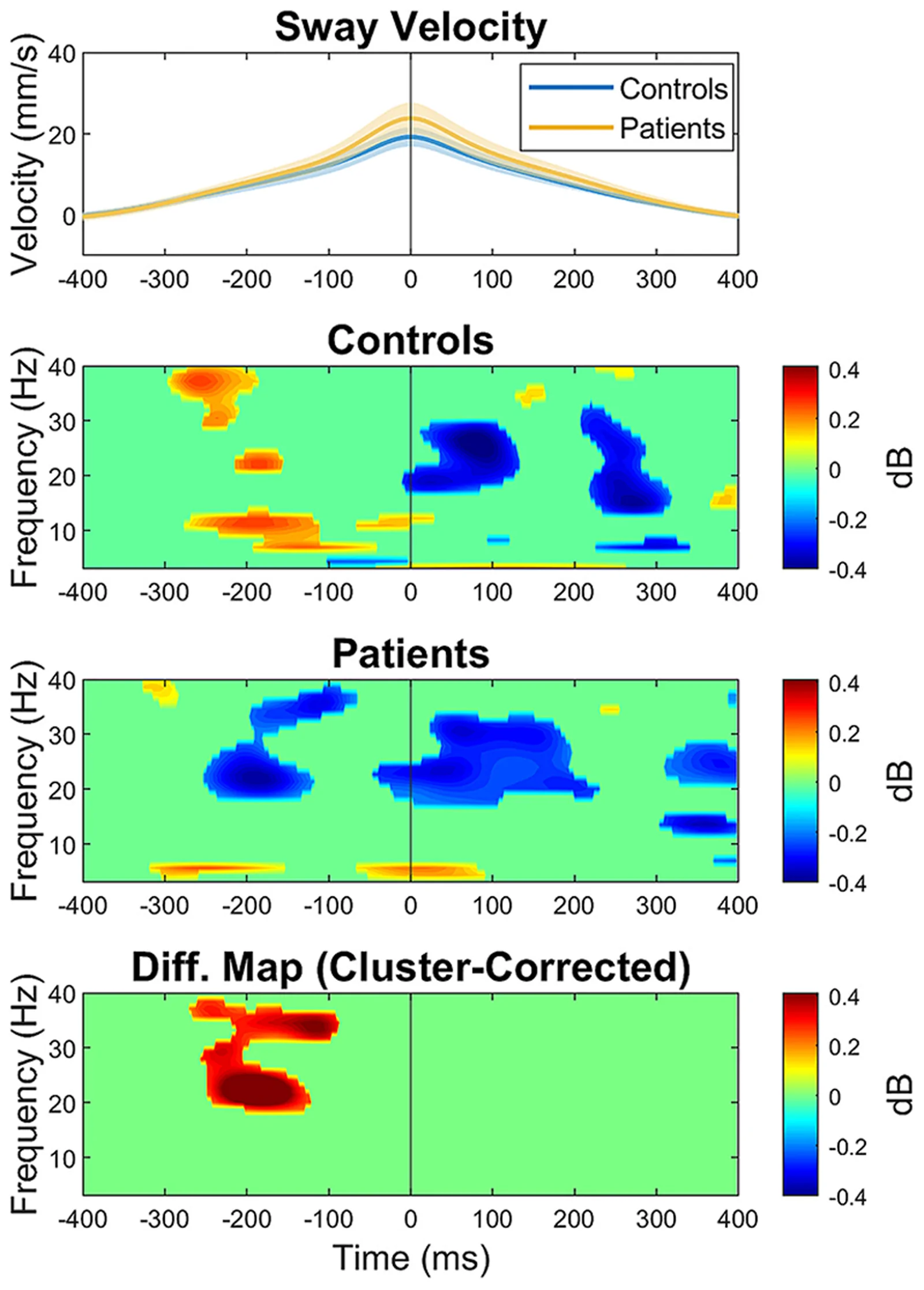
Grand-averaged sway velocity (top panel) and EEG time–frequency (ERSP) plots for Controls (second panel) and Patients (third panel) at electrode Cz, time-locked to the peak of micro-fall event (timepoint-0). Statistically significant increases in power relative to baseline are displayed in red and significant decreases in power relative to baseline are in blue (p < 0.05, permutation-based statistics). The bottom panel presents the statistically significant between-group differences (cluster-corrected to p < 0.05). As can be seen, Controls exhibited an increase in high frequency (high beta and low gamma) EEG activity between 250–150 ms prior to peak instability, whist Patients show a marked suppression in these same EEG frequency bands. Please see Supplementary Materials for these analyses repeated when removing the seven ‘unstable’ Patients (whereby identical statistically significant results were observed).

Fig. 2 presents the instability-locked averages of sway velocity (top panel), ERSP (middle panels), and cluster-corrected statistical results for ERSP analyses (bottom panel). Markedly different ERSP patterns emerged for the two groups during the early phase of micro-falls. Critically, whilst Controls exhibited statistically-significant (*p* < 0.05, permutation-based testing) phasic broadband increases in power between −250 and −150 ms prior to peak instability (as per young adults previously (Nakamura et al., 2023)), Patients exhibited a significant *decrease* in high-beta and low-gamma band power throughout the micro-fall (*p* < 0.05, permutation-based testing). This between-group early-phase difference remained statistically significant (*p* < 0.05) following cluster-based correction (see Fig. 2, bottom panel), and when removing the seven ‘unstable’ Patients (see Supplementary Materials, Fig. 1).

**Fig. 3:**
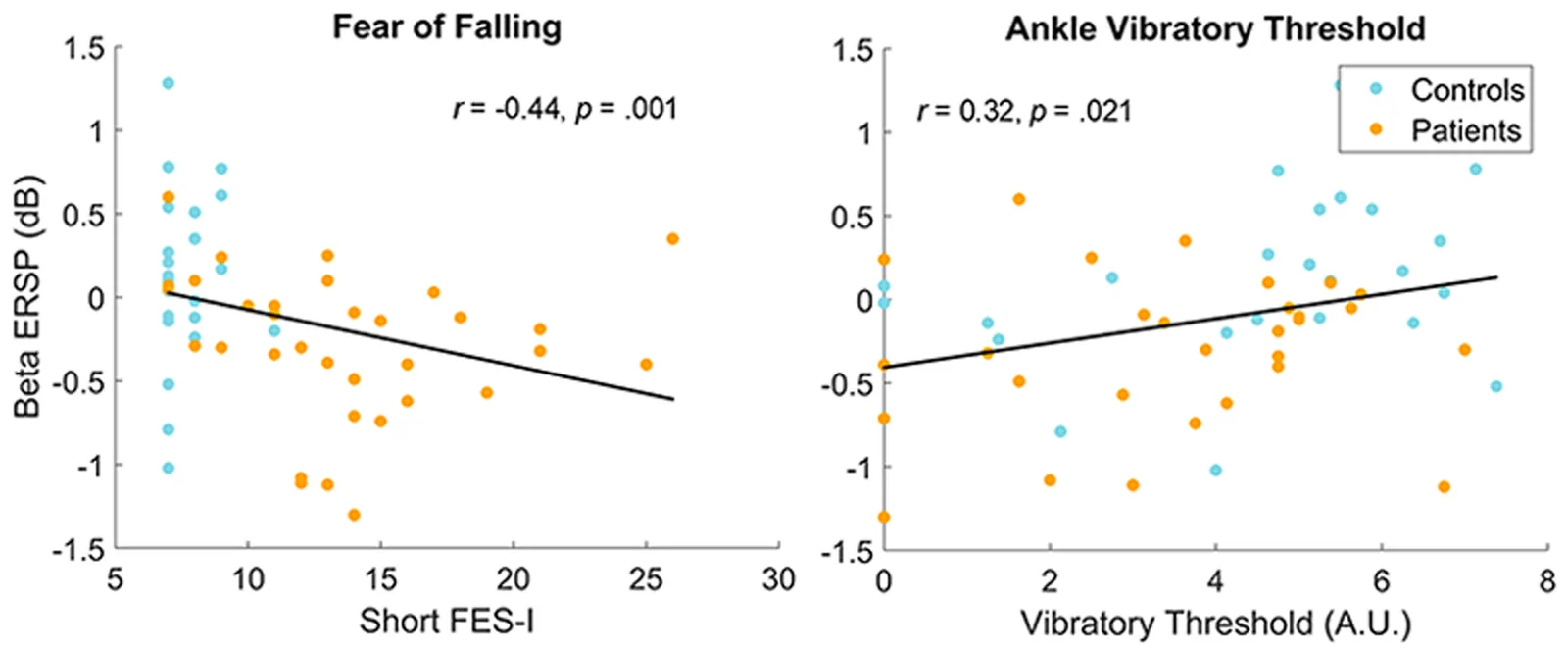
Correlations between high-beta (19–24 Hz) between −250 to −150 ms prior to peak instability and concerns about falling (short Falls Efficacy Scale-—International [FES-I]; left) and ankle vibratory perceptual thresholds (arbitrary units; right). Note, higher scores for the short FES-I indicate greater concerns (or, ‘fears’) about falling, whilst higher vibratory threshold scores indicate greater (i.e., better) ankle vibratory perception.

The most pronounced between-group differences were for high-beta (19–24 Hz) between −250 and −150 ms prior to peak instability. Averaged ERSP values within this time–frequency window were significantly correlated with concerns about falling (FES—I scores; *r* = −0.44, *p* = 0.001): Greater concerns (i.e., fear) about falling were associated with lower beta prior to peak instability (see Fig. 3). There was also evidence of a weak association between reduced beta activity and poorer ankle vibration perceptual thresholds (*r* = 0.32, *p* = 0.021; see Fig. 3). In contrast, averaged beta ERSP values were not significantly correlated with any white matter variables (WMH volumes, *r* = 0.002, *p* = 0.988; number of lesions, *r* = 0.05, *p* = 0.720; mean FA, *r* = −0.04, *p* = 0.783) or generalised anxiety (HADS; *r* = −0.14, *p* = 0.299).

## 4 Discussion

Similar to young adults previously (Nakamura et al., 2023), healthy older adults exhibited broadband increases in EEG power prior to the micro-fall peak. This was followed by strong suppression in beta activity. In contrast, dizzy older adults exhibited a marked suppression of high-frequency (beta and gamma) EEG activity *throughout* the micro-fall. These findings identify cortical beta during imbalance as a possible biomarker of chronic, idiopathic dizziness in older adults. As there were no between-group differences in balance outcomes during the quiet standing task, this rules out the possibility that the EEG results were a consequence of the patient group being more unstable (resulting in, e.g., greater movement artifacts). However, dizzy older adults reported high levels of subjective imbalance. Beta’s inhibitory role in sensory perception is well documented (Shin et al., 2017), with findings suggesting that beta events serve to decrease the relay of sensory information (Mirdamadi et al., 2024; Shin et al., 2017). We therefore propose that the high-beta activity observed prior to peak instability in healthy controls in the present work (and young adults previously (Nakamura et al., 2023)) reflects cortical inhibition of predicted sensory information related to these frequent, stereotyped and relatively small postural disturbances (explaining why these events typically occur outside of conscious awareness (Fitzpatrick et al., 1992)). This could serve to reduce higher neural processing demands, allowing unpredicted subsequent sensorimotor events (e.g., failure to arrest the micro-fall as anticipated) to be rapidly perceived and acted upon.

In contrast, dizzy older adults exhibited beta suppression prior to peak instability, which correlated significantly with measures of fear of falling. We propose that this pre-instability beta suppression reflects an anxious, top-down (i.e., ‘hypervigilant’ (Ellmers and Kal, 2024)) strategy to increase sensorimotor sensitivity to minor changes in instability and prime the motor system to respond (see (Castro et al., 2024)). However, an unintended consequence of increased sensorimotor sensitivity may be that individuals become aware of the frequent, stereotyped and relatively small micro-falls that characterise quiet stance (but typically go unnoticed (Fitzpatrick et al., 1992)), which they then perceive as ‘dizziness’. Alternatively, given the role of cortical beta in motor inhibition (Jana et al., 2020; Swann et al., 2009, 2012; Wagner et al., 2018), it is possible that beta suppression instead leads to impairments in arresting the micro-fall, and that this is why patients feel ‘dizzy’. However, an argument against this interpretation is the lack of between-group difference we observed in balance outcomes, meaning that both groups were similarly able to inhibit micro-falls. Indeed, as highlighted in the Supplementary Materials, the significant between-group differences in beta activity also remained when removing the seven patients who were markedly more unstable than controls. Nonetheless, postural control may have been characterised by subtle differences in sensorimotor strategy (e.g., altered cortico-muscular control) that the present analysis was unable to detect.

Whilst previous work has reported associations between fear of falling and idiopathic dizziness in older adults (Castro et al., 2024), the present findings enhance our mechanistic understanding of this relationship. Specifically, they imply that fear may increase sensorimotor sensitivity to (and conscious awareness of) minor postural disturbances. The present findings also identify beta suppression during micro-falls as a possible biomarker of fear of falling and associated top-down regulation of balance. However, future work is needed to confirm this interpretation. Due to the correlational nature of the present analysis, it is equally plausible that disrupted sensory processing (see the significant, albeit weak, correlations between ankle vibratory perceptual thresholds and beta activation) leads to both alterations in sensorimotor control and fear of falling – rather than the other way around. Future work should therefore test the existence of a causal relationship between fear of falling and decreased beta activity through, for instance, experimentally inducing postural threat in older adults (see (Ellmers et al., 2022, 2021; Johnson et al., 2019)).

The present findings build on previous work in this journal that focused on averaged EEG outcomes in older adults with idiopathic dizziness (rather than time–frequency decomposition during discrete instances of instability, as in the present work). For instance, Ibitoye et al. (2021) described altered low-frequency (delta) EEG loops in dizzy older adults – which related to white matter MRI outcomes (unlike our findings which correlated with fear of falling only). The causes of idiopathic dizziness in older adults are multifactorial, and include interacting physiological (e.g. subclinical small vessel disease and balance impairments) and subjective (e.g. fear of falling) factors (Castro et al., 2024). While we found no association between EEG findings and white matter MRI outcomes, previous work has reported strong links between small vessel disease and idiopathic dizziness in older adults (Ahmad et al., 2015; Castro et al., 2024; Ibitoye et al., 2022, 2021; Liu et al., 2024). However, our present findings suggest that there may be an additional *indirect* route: any factor that makes an older person concerned or fearfull about falling (including subtle disturbances in balance from subclinical small vessel disease (Castro et al., 2024)) could influence symptoms through disruptions to the sensorimotor control of the frequent micro-falls that characterise quiet stance. Future studies should look to confirm these interpretations through experimental manipulation of fear of falling, whilst also addressing the limitations of the present work including the limited focus on a single EEG channel (rather than high-density EEG that can be localised to different neural sources/brain areas (Huang and Ferris, 2023)) and absence of any neuromuscular (i.e., EMG) recordings.

## 5 Conclusions

In conclusion, we found disruptions to the cortical control of micro-falls in older adults with idiopathic dizziness, with these patients exhibiting decreased beta activity during imbalance compared to healthy controls. We propose that these alterations may reflect a conscious, top-down (i.e., hypervigilant) strategy to increase sensorimotor sensitivity to postural disturbances, which paradoxically causes persistent feelings of subjective imbalance (Castro et al., 2024). Future work should look to specifically test these interpretations.

## Appendix A. Supplementary data

Supplementary data to this article can be found online at https://doi.org/10.1016/j.clinph.2025.03.032.

## Figures and Tables

**Table 1:** Demographic details of the two groups.

Variable	Controls (N = 25), Mean (SD)	Patients (N = 33), Mean (SD)	p value
Age (years)	76.9 (6.0)	77.3 (6.4)	0.803
Gender (female, %)	56.0 %	60.6 %	0.742
Height (cm)	167.7 (10.2)	169.6 (9.6)	0.481
Weight (kg)	67.2 (11.3)	74.7 (18.1)	0.197
Concern about falling (Short FES-I, 7–28)	7.6 (1.0)	13.9 (4.8)	<0.001
Anxiety (HADS-A, 0–21)	2.8 (2.3)	6.6 (4.4)	0.001
Depression (HADS-D, 0–21)	2.7 (3.1)	6.3 (3.7)	<0.001
Dizziness handicap (DHI, 0-100)	0.2 (0.7)	33.6 (18.7)	<0.001
Short Physical Performance Battery (0–12, higher = better)	11.2 (1.0)	9.7 (2.1)	0.003
Ankle vibration perceptual threshold (0–8, arb. units; higher = better)	4.50 (2.17)	3.48 (2.02)	0.051
WMH (volume/ml)*	9.36 (9.02)	14.41 (19.42)	0.441
Number of lesions*	13.71 (8.47)	14.32 (6.12)	0.764
Mean FA (lower values suggest reduced white matter structural integrity)*	0.439 (0.061)	0.442 (0.055)	0.865

FES-I = Falls Efficacy Scale—International; HADS-A = Hospital Anxiety and Depression Scale, Anxiety; HADS-D = Hospital Anxiety and Depression Scale, Depression; DHI = Dizziness Handicap Inventory; VSS = Vertigo Symptoms Scale; WMH = white matter hyperintensity; FA = fractional anisotropy.

* Note, MRI data were only available for 24 Controls and 28 Patients.
